# Radionuclides Transfer from Soil to Tea Leaves and Estimation of Committed Effective Dose to the Bangladesh Populace

**DOI:** 10.3390/life11040282

**Published:** 2021-03-27

**Authors:** Nurul Absar, Jainal Abedin, Md. Mashiur Rahman, Moazzem Hossain Miah, Naziba Siddique, Masud Kamal, Mantazul Islam Chowdhury, Abdelmoneim Adam Mohamed Sulieman, Mohammad Rashed Iqbal Faruque, Mayeen Uddin Khandaker, David Andrew Bradley, Abdullah Alsubaie

**Affiliations:** 1 Department of Computer Science and Engineering, BGC Trust University Bangladesh, Chittagong 4381, Bangladesh; nabsar@bgctub.ac.bd (N.A.); abedinj7110@bgctub.ac.bd (J.A.); 2 Hughes Network Systems, 11717 Exploration Lane, Germantown, MD 20876, USA; mashiur.rahman@hughes.com; 3 Department of Physics, University of Chittagong, Chittagong 4331, Bangladesh; mhmiah@cu.ac.bd (M.H.M.); snaziba@yahoo.com (N.S.); 4 Atomic Energy Centre-Chittagong, Radioactivity Testing and Monitoring Laboratory, Bangladesh Atomic Energy Commission, Chittagong 4209, Bangladesh; masud.kamal@gmail.com (M.K.); mantaz54@yahoo.com (M.I.C.); 5 Department of Radiology and Medical Imaging, College of Applied Medical Sciences, Prince Sattam Bin Abdulaziz University, P.O. Box 422, Alkharj 11942, Saudi Arabia; a.sulieman@psau.edu.sa; 6 Space Science Centre (ANGKASA), Universiti Kebangsaan Malaysia, Bangi 43600, Malaysia; rashed@ukm.edu.my; 7 Centre for Applied Physics and Radiation Technologies, School of Engineering and Technology, Sunway University, Bandar Sunway 47500, Malaysia; d.a.bradley@surrey.ac.uk; 8 Centre for Nuclear and Radiation Physics, Department of Physics, University of Surrey, Guildford, Surrey GU2 7XH, UK; 9 Department of Physics, College of Khurma, Taif University, P.O. Box 11099, Taif 21944, Saudi Arabia; a.alsubaie@tu.edu.sa

**Keywords:** soil, tea leaves, HPGe γ-ray spectrometry, terrestrial and anthropogenic radionuclides, threshold consumption rate, committed effective dose

## Abstract

Considering the probable health risks due to radioactivity input via drinking tea, the concentrations of ^226^Ra, ^232^Th,^40^K and ^137^Cs radionuclides in the soil and the corresponding tea leaves of a large tea plantation were measured using high purity germanium (HPGe) γ-ray spectrometry. Different layers of soil and fresh tea leaf samples were collected from the Udalia Tea Estate (UTE) in the Fatickchari area of Chittagong, Bangladesh. The mean concentrations (in Bq/kg) of radionuclides in the studied soil samples were found to be 34 ± 9 to 45 ± 3 for ^226^Ra, 50 ± 13 to 63 ± 5 for ^232^Th, 245 ± 30 to 635 ± 35 for ^40^K and 3 ± 1 to 10 ± 1 for ^137^Cs, while the respective values in the corresponding tea leaf samples were 3.6 ± 0.7 to 5.7 ± 1.0, 2.4 ± 0.5 to 5.8 ± 0.9, 132 ± 25 to 258 ± 29 and <0.4. The mean transfer factors for ^226^Ra, ^232^Th and ^40^K from soil to tea leaves were calculated to be 0.12, 0.08 and 0.46, respectively, the complete range being 1.1 × 10^−2^ to 1.0, in accordance with IAEA values. Additionally, the most popularly consumed tea brands available in the Bangladeshi market were also analyzed and, with the exception of ^40^K, were found to have similar concentrations to the fresh tea leaves collected from the UTE. The committed effective dose via the consumption of tea was estimated to be low in comparison with the United Nations Scientific Committee on the Effects of Atomic Radiation (UNSCEAR) reference ingestion dose limit of 290 μSv/y. Current indicative tea consumption of 4 g/day/person shows an insignificant radiological risk to public health, while cumulative dietary exposures may not be entirely negligible, because the UNSCEAR reference dose limit is derived from total dietary exposures. This study suggests a periodic monitoring of radiation levels in tea leaves in seeking to ensure the safety of human health.

## 1. Introduction

The most common forms of ionizing radiation on earth, resulting from terrestrial, extra-terrestrial and anthropogenic sources, are α- and β-particles and γ-rays [1,2]. According to the National Council on Radiation Protection and Measurements (NCRP), environmental radiation is the most significant source of radiation exposure to humans [3]. Interestingly, although the International Atomic Energy Agency (IAEA) reported that the public exposure from natural radiation is of little health concern [4], the World Nuclear Association (WNA) states that any dose of radiation involves a possible risk to human health [5]. Since ^238^U and ^232^Th decay series and singly occurring ^40^K are the most abundant radionuclides found naturally in soil, air, water, rocks, plants and foodstuffs [5], to protect the public against unwanted exposures to natural radiation, the radioactivity in environmental samples, including foodstuffs, needs to be monitored periodically [4].

The surface soil, especially the top layer in the earth, is a mixture of various components in the natural environment [6,7]. Although the main source of U, Th and K is the earth crust, their contents appear at varying levels in the soils of different regions in the world following the variation of the local geology [8]. In addition to the prevailing concentration of terrestrial radionuclides in acidic soils, at high altitudes the contribution from extra-terrestrial radioactivity may be enhanced, moreover with fallout of artificial radionuclides that may be subject to greater deposition. The latter concerns atmospheric nuclear device testing or unplanned phenomena such as the Three Mile Island power plant accident, the Chernobyl accident and the Fukushima Dai-ichi nuclear power plant accident [9,10,11,12]. Regardless of origin (i.e., natural or artificial), the radionuclides may appear in plants along with the uptake of minerals and nutrients required for their vitality, majorly by the root system [13]. Their availability in plant life enables them to be transported to humans via the daily intake of foodstuffs [14,15,16]. Thus, it is necessary to know the natural radioactivity in a particular area to obtain their distribution, modelling and transport processes leading to the estimation of radiation dose and hazards to the general population [6].

Due to its pleasant taste, aroma and positive physiological functions, tea is one of the most popular stimulating drinks in the world after water [17,18,19]. It is generally obtained by processing the leaves and leaf buds of the Camellia sinensis plant. Primarily there are four types of processed teas: green, black, oolong and brick tea [20]. Green tea is obtained by drying and roasting the tea leaves without any fermentation, whereas an additional fermentation produces black tea leaves. A partial fermentation of tea leaves results in oolong tea. Brick tea is made from the blocks of whole or finely ground black tea, compressed in a form under extremely high pressure. Asian populations generally drink the semi-fermented green tea and fermented black teas [21] as a hot drink. While, at present, tea is cultivated in more than 40 countries in the world, the major portion (90%) is produced by Asian countries [22]. Due to the presence of biologically active compounds (antitoxin, antioxidant, anti-inflammatory, antibacterial, antiviral, anti-carcinogenic, etc.) like polyphenols, amino acids and vitamins in tea [23,24], tea drinking has been promoted for centuries [25,26,27]. Controversies about the benefits and risks due to the consumption of tea are not completely absent, but the few reported toxic effects are outclassed by its countless health-promoting benefits [28]. Harmful effects like stomach ache, intestinal gas, heartburn and abdominal pain from over consumption of tea are identified due to the presence of caffeine, aluminum and the influence of tea polyphenols on iron bioavailability [29].

Following the suitable geographical location and weather conditions for tea plantation, tea plantations were established in the hilly areas of Sylhet, Moulavibazar, Comilla and Chittagong regions in Bangladesh, centuries ago [30]. Moreover, due to recent developments in socio-economic conditions, tea consumption is increasing at a significant level among Bangladeshi population [31]. In producing more than 95 million kg of tea in 2019, harvested from about 115,757 hectares of land, Bangladesh has become the world’s tenth largest tea producer and the world’s ninth tea exporting country [32]. As a member of Bangladesh Tea Board and Bangladesh Tea Association, the Udalia Tea Estate (UTE), since 1962, has played a very important role in quality tea production. In recent times, the UTE has come to be ranked highly among the existing tea estates in Bangladesh. In meeting the growing domestic demand for premium black and green tea as well as that of international markets, UTE has secured a position not only as a tea producer but also as a multi-product estate. In particular, it enjoys abundant rainfall, is in an area of elevated altitude above sea level and has acidic and well-draining soils, all combining to make favorable conditions for tea production.

Since plants produce their necessary energy via the use of leaves together with the photosynthesis process, the leaves may have more activity than the other parts of plants, therefore they may contain relatively more radionuclides which are normally taken up by the root system along with other minerals. Moreover, leaves are more exposed to aerial deposition, e.g., radionuclides in dust or radionuclide particles themselves, if there are any artificial phenomena in the surrounding environment or nearby countries. Therefore, tea plants may be subjected to direct and indirect contamination of various radionuclides, and these radionuclides can be distributed in different parts of the plants according to the chemical characteristics and parameters of the plants and soil [33]. Since tea forms the second most popular drink (after water) in all populations in Bangladesh, the presence of a low level of radioactive material in tea leaves may produce a non-negligible health hazard via cumulative exposures. By acknowledging that ingestion doses above permissible levels are harmful for human beings [34], assessment of radionuclides due to the consumption of foodstuffs is important for public health. Furthermore, assessment of any release of radioactivity to the environment is important for the protection of public health, especially if the released radioactivity can enter into the food chain [35].

While studies of natural radioactivity in various foodstuffs are available in the literature, information on the distribution and enrichment of radionuclides in tea leaves is sparse, especially in tea leaves collected from the major tea gardens including the UTE in Bangladesh. The main objectives of the present study are to determine the transfer of radionuclides from soil to tea leaves harvested from the Udalia Tea Estate, further calculating the associated health hazards following the consumption of tea by the populace in Bangladesh. The activity concentrations of marketed tea leaves were also analyzed to observe the effect of manufacturing processes. This study may also help to enrich the radioactivity database on tea, i.e., the most popular stimulating drink.

## 2. Experimental

### 2.1. Local Geology of the Udalia Tea Estate

**Figure 1:**
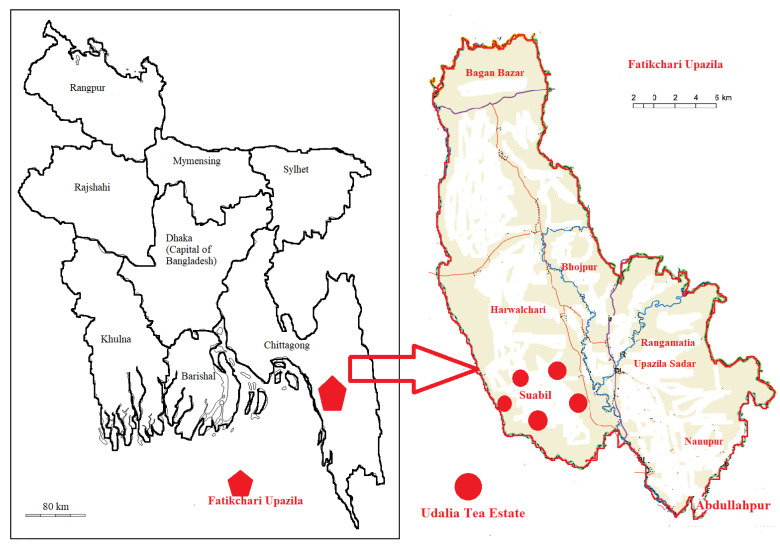
Location of the Udalia Tea Estate at the Fatikchari Upazila of the Chittagong district in Bangladesh.

The Udalia Tea Estate (UTE) is located in the hilly region of Fatickchari Upazilla in the Chittagong district of Bangladesh (see in Figure 1). The UTE can be addressed as latitude 22°36′39″–22°39′41″ N and longitude 91°45′6″–91°51′15″ E. The estate is covered by low hill ranges and terraces having an altitude of 30–46 m above sea level and contains an area of 3096 acres (3.9 km × 3.2 km = 12.5 square kilometer) [36]. The climate of this area is tropical monsoon. An average annual rainfall of about 2794 mm is recorded in this area, while July is the wettest month. The soils in this area are mainly yellowish to reddish brown, the texture is mostly clay loam on flat land while the hilly soil is mostly sandy loam to coarse sand, which is characterized by broken shale or sandstone and mottled sand at different depths [37]. The soils are strongly acidic and poor in organic matter and nutrients. 

### 2.2. Sample Collection and Preparation

The samples (soil and tea leaves) were collected from different places in the Udalia Tea Estate (UTE). The sampling location was chosen on a random basis, but the distance between each sampling location was almost 700–800 m. Since the estate consists of ranges of low lying hills, separate hills were chosen as different sampling locations. A total of 5 locations were chosen for collecting the tea leaves and the surrounding soil samples throughout the garden. While an approximate amount of 2.5 kg of fresh tea leaf samples were collected from each selected location around the UTE, a total of 4 (×3) soil samples were also collected from three different depths, 0 to 5 cm, 6 to 12 cm and 13 to 20 cm, of the corresponding locations to the tea leaves samples. More specifically, the soil samples were collected from four different points within an area of 1 m^2^ around the tea tree. The 25-year-old tea trees were selected for collection of the tea leaf samples. Usually, the branches of the tea plant are cut and are fertilized twice per year, and in some cases fertilizers are used more than twice for influencing the growth of the tea plant in the garden. At present, the UTE produces approximately 0.7 million tons of tea per year with six separate grades of tea including the export quality one. In the local market and throughout the country, this estate supplies one of the popular tea brands “Mostafa tea”. Five marketed tea leaf samples were also collected from the local market, allowing comparison of the measured radioactivity from these with that from the fresh tea leaves collected from the UTE. 

The procedure for sample collection followed that recommended by the IAEA [4]. The collected samples were stored separately in sealed plastic bags and tagged with an identification number, and with the date and location of sampling. The samples were dried under direct sunlight for several days to allow evaporation of moisture content, subsequently being further dried for a period of 24 h in an oven maintained at 85 °C to remove any remaining moisture. The dried samples were then mechanically crushed into a fine powder, homogenized with a mortar and pestle and filtered through a sieve of 0.395 mm mesh size to obtain similarly sized particles. A constant dry weight was measured out for each sample evaluation. For the determination of activity concentration, the dried sample was transferred to an individual cylindrical container having dimension of 3.5 cm height and 8.5 cm diameter. To settle and obtain a homogeneous mixture of the samples, the containers were simply shaken by hand. The containers were then sealed tightly by using an insulating tape to reduce the possibility of moisture contamination. The samples were then kept undisturbed for 5–7 weeks at room temperature to attain secular equilibrium between short-lived progeny with the respective long-lived parents, ^226^Ra (from ^238^U) and ^228^Ra (from ^232^Th) [38,39]. It was assumed that ^222^Rn and ^220^Rn could not escape from the sealed containers during the period of storage. The samples were then ready for subsequent measurement and analysis by γ-ray spectrometry.

### 2.3. Measurement of Radionuclides

**Figure 2:**
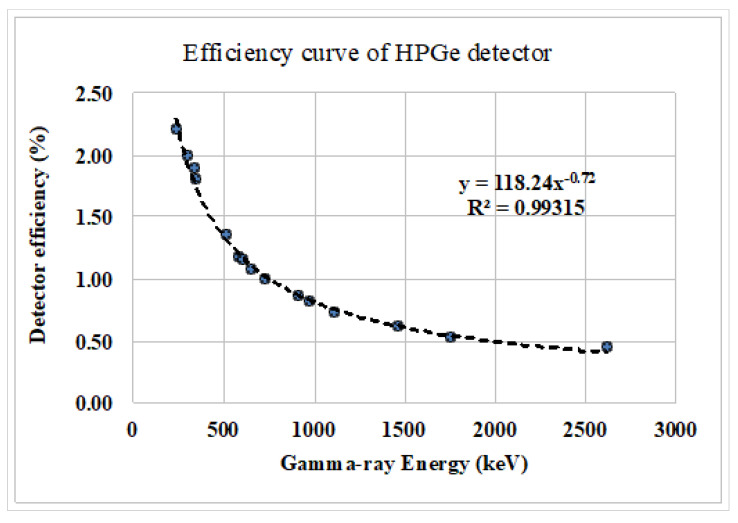
Counting efficiency curve of the HPGe (high-purity germanium) detector.

This study used a co-axial high-purity germanium (HPGe) γ-ray detector (GC2018, CANBERRA, USA), having a relative efficiency of 20%, resolution of 1.8 keV at 1332 keV of peak of ^60^Co source, to measure the samples and standards obtained from the IAEA. The detector was coupled with a digital spectrum analyzer and GENIE 2000 to acquire the γ-ray spectra emitted from the samples. To ensure a low background environment, a cylindrical lead shielded arrangement (5.08 cm thick) with fixed bottom and movable cover was installed. The efficiency of the detector was measured using the reference samples RGU-1, RGTh-1 and RGK-1 provided by the IAEA [40], with results as presented in Figure 2. The standard sources containing known concentrations of ^226^Ra, ^232^Th and ^40^K were supplied by the Canada Center for Mineral and Energy Technology (CAMET) under a contract with the IAEA. Considering the leaves’ texture and density, a radioactive standard with leafy vegetables was prepared by mixing/spiking ^226^Ra standard source of solid matrices in identical containers to the samples, and using them accordingly. Necessary information on the calibration of the efficiency of the detector is available elsewhere [41]. In this study, each sample was measured for 10,000 seconds to achieve reasonable statistics. The net count rate from the primordial radionuclides originating from the samples was obtained by subtracting the background count from the gross count, both acquired for the same counting time. The activity concentrations of ^226^Ra and ^232^Th radionuclides were assessed using the characteristic gamma lines of their short-lived progeny [42,43]. The concentrations of ^40^K and ^137^Cs were determined by the gamma ray lines of 1460.77 keV and 661 keV, respectively. For evaluation of ^226^Ra and ^232^Th, a weighted mean approach was applied following reference [14].

### 2.4. Calculation of Activity Concentration

Activity concentrations of radionuclides (Bq kg^−1^) in surface soil, sub-surface soils and plant samples were calculated using Equation (1) [41]:

(1) $$\mathrm{Activity}\;\mathrm{concentration}\;=\;\frac{\mathrm{CPS}\times 100\times 1000}{\varepsilon_{f}\left(\%\right).\times I_{\gamma }\times w_{s}(\mathrm{kg})}$$

where CPS represents the net counts per second, *ε_f_* the efficiency of the detector, *Iγ* the branching ratio and *W_s_* the weight of the sample in kg. The statistical uncertainties were expressed in terms of standard deviation (±σ), where σ is expressed in Equation (2) [41]:

(2) $$\sigma =\sqrt{\left[\frac{N_{s}}{T_{s}^{2}}+\frac{N_{b}}{T_{b}^{2}}\right]}$$

where *N_s_* and *N_b_* represent the sample and background counts in time *T_s_* and *T_b_*, respectively. The total uncertainty for each measured sample was calculated taking into account the statistical and other components of uncertainty. The combined uncertainty of the activity was estimated by using the quadratic sum of relevant quantities, which can be defined by Equation (3) [44]:

(3) $$\Delta A=A\times \sqrt{{\left(\frac{\Delta N}{N}\right)}^{2}+{\left(\frac{\Delta \varepsilon_{\gamma }}{\varepsilon_{\gamma }}\right)}^{2}+{\left(\frac{\Delta \rho_{\gamma }}{\rho_{\gamma }}\right)}^{2}+{\left(\frac{\Delta m_{s}}{M_{s}}\right)}^{2}{}^{}}$$

where Δ*A* is the combined uncertainty of each measured value. The symbols Δ*N*, Δ*ε_γ_*, Δ$\rho_{\gamma }$ and Δ*m_s_* represent the uncertainties due to the counting statistics, *N* (<7%), detection efficiency, *ε*_γ_ (4%), gamma ray emission probability, $\rho_{\gamma }$(<1%), and sample weight, *M_S_* (<2%), respectively. The determined radioactivity levels, together with the uncertainties, are presented in Table 1.

The lower limit of detection or the minimum detectable activity concentration (MDA) of the measurement system was calculated using Equation (4) [45,46]:

(4) $$\mathrm{MDA}=\;\frac{k_{\alpha }\times \sqrt{\beta }}{\varepsilon_{\gamma }\times \rho_{\gamma }\times T_{s}\times M_{s}}$$

where *K_α_* represents the statistical coverage factor which is equal to 1.64 (at the 95% confidence level), *β* is the background count in the energy of interest and the other symbols *ε*_γ_, $\rho_{\gamma }$, *T_S_* and *M_S_* represent detection efficiency, gamma-ray emission probability, counting time, and sample weight, respectively. The MDAs for the studied radionuclides ^226^Ra, ^232^Th, ^40^K and ^137^Cs were calculated to be 0.32 Bq kg^−1^, 0.60 Bq kg^−1^, 2.5 Bq kg^−1^ and 0.4 Bq kg^−1^, respectively.

### 2.5. Soil to Tea Leaves Transfer Factor (TF)

Within the food we eat, plants are the principal recipients of radioactive contamination, a result of atmospheric or other releases of radionuclides and from naturally occurring radioactivity within the soil [40]. Basically, the transfer factor defines the uptake of radionuclides from soil to plants, which can be calculated by the ratio of the radioactivity per unit dry weight of plant (*C_P_*) to the radioactivity per unit dry weight of soil (*C_S_*) in the rooting zone, using the Equation (5) [47,48]:

(5) $$\mathrm{TF}=\frac{C_{P}}{C_{S}}$$

Dry weight analysis is preferred, the amount of radioactivity per kilogram dry weight being subject to much less variability than the amount per unit fresh weight, thereby reducing uncertainties in the measured TF (transfer factor) [49]. The calculated TFs for the studied tea leaf samples are shown in Table 1.

### 2.6. Annual Committed Effective Dose (ACED)

Following the consumption characteristics of foodstuffs, the committed effective dose due to the ingestion of radionuclides can be calculated using Equation (6), as below [50]:

(6) $$\mathrm{AECD}\;\left(\mu \mathrm{Sv}/y\right)=C_{r}\times \overset{3}{\underset{i=1}{\sum }}D_{cfi\;}\;\times \;A_{i}$$

where *C_r_* is the intake of radionuclides through use of the tea leaves, *D_CF,i_* are the ingestion dose conversion coefficients of 2.8 × 10^−7^ Sv Bq^−1^, 2.2 × 10^−7^ Sv Bq^−1^ and 6.2 × 10^−9^ Sv Bq^−1^ for ^226^Ra, ^232^Th and ^40^K, respectively, for an adult [51] and A_i_ is the measured activity concentration (Bq.kg^−1^) of each radionuclide. According to the typical statistics, an average of 2 g of tea leaves is needed to prepare a cup of tea and if one person drinks two cups of tea per day, then an amount of some 1.5 kg/year is consumed by an individual. The two cups of tea is a typical tea consumption characteristic for the Bangladeshi population. The *C_r_* is also defined as the consumption rate.

### 2.7. Threshold Consumption Rate of Tea (kg/y)

The threshold consumption rate (*DI_thresh_*) represents a reference dietary level to avoid deleterious health hazards due to the intake of radionuclides via foodstuffs [52]. The particular threshold data due to the drinking of tea can be estimated by using the following Equation (7) [53]:

(7) $$DI_{thresh}\;(\mathrm{kg}/y)\;=\;\frac{E_{ave}}{\sum_{i=1}^{3}D_{cfi\;}\;\times \;A_{i}}$$

were *E_ave_* (290 μSv/y) is the threshold committed effective dose due to the ingestion of radionuclides of interest via the consumption of foodstuffs [54,55], A_1_, A_2_ and A_3_ are the activity concentrations of ^226^Ra, ^232^Th and ^40^K, respectively, in the tea leaf samples and *D_cfi_* is the activity to dose conversion coefficient for the radionuclides of interest, as before.

### 2.8. Carcinogenic Risk

The carcinogenic risk for a population is estimated by assuming a linear no threshold, dose–effect relationship as per ICRP practice. For low doses, the ICRP suggest a fatal cancer risk factor of 0.05 Sv^−1^ [56], which indicates that the probability of a person dying of cancer is increased by 5% for a total dose of 1 Sv received during a lifetime. The estimated average annual committed effective dose for tea leaves is used herein to calculate the carcinogenic risk for an adult, made of the following relationship Equation (8):

(8) $$ElCR=\mathrm{AECD}\left(\mu Sv/y\right)\;\times R_{f}\left(Sv^{-1}\right)\times A_{ls}\left(y\right)$$

where *R_f_* is the risk factor per sievert of annual effective dose received by the consumption of tea and *A_ls_* is the cumulated time of tea consumption by Bangladeshi populace. Considering the local typical tea consumption characteristics, a duration of 50 years was used for both sexes.

## 3. Results and Discussion

### 3.1. Activity Concentration in the Tea Garden Doil Samples

The mean activity concentrations of ^226^Ra, ^232^Th, ^40^K and ^137^Cs in the soil samples collected from five locations of UTE are given in Table 1. The measured values in the investigated soil samples are found in the order ^40^K > ^232^Th > ^226^Ra > ^137^Cs. ^40^K dominates over the other nuclides, which is not unexpected. This is because potassium is the seventh most abundant element in the Earth’s crust, making up 2.6% of the weight of the earth’s crust [57]. The greater activity concentration of ^232^Th over that of ^226^Ra may be attributed to the differences in the physical and chemical characteristics in a natural environment. In the earth’s crust, both uranium and thorium tend to occur together due to the some inherent characteristics. However, throughout the various superficial processes like weathering and transportation, and the soil characteristics (pH and redox), they become fractionated. In general, thorium possesses low solubility and accumulates on particular phases whereas uranium is chemically more soluble and mobile. Consequently, uranium can easily be redistributed and transported in various environmental matrices compared to thorium [48].

Table 1 shows the mean activity concentrations of ^226^Ra in soil samples for all locations other than that at location 2 to be greater than the UNSCEAR reported worldwide mean value of 35 Bq.kg^−1^ [49]. Among the three studied layers/depths of soil, the greatest concentration of ^226^Ra (53 ± 8 Bq.kg^−1^) was at location 5, at a depth of 13- to 20 cm, whereas, the minimum concentration of ^226^Ra (27 ± 7 Bq.kg^−1^) was at location 2, at a depth of 6 to 12 cm. This may be correlated to the ambient environment, i.e., the presence of high moisture content in the clay silty sand soil of this location which allows better solubility of ^226^Ra [58].

For all locations the mean activity concentrations of ^232^Th are greater than the worldwide mean value of 30 Bq.kg^−1^ [49], while the ^40^K data for all locations other than location 2 are less than the UNSCEAR [49] reported mean concentration of 400 Bq.kg^−1^. In respect to the vertical distribution, the greatest concentration of ^232^Th (82 ± 11 Bq.kg^−1^) was in soil from location 4, at a depth of 6 to 12 cm, whereas the minimum ^232^Th concentration (29 ± 7 Bq.kg^−1^) was from soil at location 5, at a depth of 0 to 5 cm. Soil samples from location 5 are clay silty sand and have large carbonate content, a matter correlating with the low ^232^Th concentration. The data show the level of natural radioactivity forming a similar distribution in the surface and deep layered soils.

The greatest concentration of ^40^K (672 ± 81 Bq.kg^−1^) was shown to be at location 2, at a depth of 6 to 12 cm, whereas the minimum ^40^K concentration (201 ± 78 Bq.kg^−1^) was at location 1, at a depth of 6 to 12 cm. The majority of ^40^K is a part of a clay mineral component rather than organic matter and its mobility depends on its solubility in the soil [59]. The low concentration of ^40^K may be correlated to the soil texture, i.e., the presence of more sandy soil. Moreover, use of NPK fertilizer at least two times per year for better yield of leaves may contribute to the higher values of ^40^K activity [60,61].

^137^Cs, an anthropomorphic nuclide, as detected in trace amounts in the UTE soil, predominantly in the topsoil layers and less so or otherwise not detectable in sub-surface layers. The greatest ^137^Cs mean activity concentration, at 10 ± 1 Bq.kg^−1^, was found in surface soil at location 1, at a depth of 0 to 5 cm, while the lowest concentration of 3 ± 1 Bq.kg^−1^ was found at location 5, at the same depth. ^137^Cs in other locations was not detected, the one exception being at location 1, at a depth of 6 to 12 cm. The mean ^137^Cs concentrations at the different locations were found to be less than the world average value 51 Bqkg^−1^ as reported by UNSCEAR [49]. The ^137^Cs is a quasi-permanent source of external gamma ray exposure, the activity slowly decaying in accordance with a half-life of some 30 years. The small likelihood that this nuclide will form a significant soil to plant pathway is generally acknowledged, the contaminant for the most part being linked to widely publicized nuclear establishment accidents. When detected, most typically at very low levels, the variation in the activity concentrations of the radionuclides are due to meteorological factors, the difference in sampling depth, physiochemical soil characteristics and the time of deposition.

### 3.2. Activity Concentration in Tea Leaf Samples

The measured activity concentrations of ^226^Ra, ^232^Th, ^40^K and ^137^Cs radionuclides in tea leaf samples collected from the Udalia Tea Estate as well as from the local market are summarized in Table 1. The concentrations of radionuclides in tea leaf samples are reported in Bq.kg^−1^ dry weight. The activity concentrations of studied radionuclides in the investigated tea leaf samples were in the order ^40^K > ^226^Ra > ^232^Th > ^137^Cs. The activity concentrations of ^226^Ra were found to be greater than that of ^232^Th in most of the tea leaf samples collected from UTE. One probable reason is that the ^238^U (^226^Ra) tends to move towards the outer extremities of the tree and accumulates more greatly in new leaves and sprouts [62].

The greater concentration of ^40^K in tea leaf samples can be attributed for the most part to the specific metabolic processes of potassium involved in plant growth. Furthermore, for faster plant growth, the extra use of muriate (potassium chloride) of potassium fertilizer may be another factor causing the increase of ^40^K concentration in the tea leaf samples [60]. It has been reported that about 88–96% of K is taken up by the plant from the soil through the root system [61]. Since plants take up a high amount of potassium and natural potassium contains 0.0117% of ^40^K, the detection of high level of ^40^K in plants is not unexpected.

### 3.3. Transfer Factors (TF) of Radionuclides from Soil-to-Tea Leaf

Soil to tea leaf transfer factor (TF) values from the five different locations are also presented in Table 1, the values depending on soil properties such as nutrient and moisture contents and pH [51]. It can be observed from the results that the TF values for ^226^Ra, ^232^Th and ^40^K lie within the range 1.1 × 10^−2^ to 1.0, in accordance with values reported by the IAEA [63]. ^137^Cs in all of the tea leaf samples was found to be below the detection limit, therefore the transfer factors could not be calculated. Note that the IAEA report a TF range of 0.02–3.2 for ^137^Cs [63]. This indicates that, compared to ^226^Ra, ^232^Th and ^40^K, ^137^Cs is less efficiently transported from soil to the tea bush, as well as to the leaves 

The transfer factors in the studied tea leaves are in the order ^40^K > ^226^Ra > ^232^Th > ^137^Cs, that for ^40^K being significantly greater than those of other radionuclides in all samples. It is well known that K is a very essential nutrient for plants metabolism and depending upon the particular metabolism a variable amount of K is taken up by plants from soil. Since elemental potassium is homeostatically controlled by the body (intake and excretion maintaining balance), such amounts of ^40^K in tea leaves are not to be considered to be of any particular concern as a potential radiation hazard. The actual concentration of radium in plant species clearly depends on the radium content of soil, its uptake by the plants species and also the metabolic characteristics of the plants [14]. Moreover, the chemical factors such as the presence of exchangeable amount of calcium in the soil may influence the absorption rate of radium by the plants [64]. The calculated TF show mean values of less than 1 for all radionuclides. It is worth mentioning that a value of TF >1 is indicative of radiation hazards to human health via the soil–plant–human body pathway. On the other hand, a TF = 1 would be indicative of a particular species or plant forming a useful natural process for decontamination of soil affected by a nuclear accident or deliberate nuclear device testing.

Table 2 shows a comparison of results from the present study with reported data for tea leaves from the Chittagong region of Bangladesh. Considering the similar geographical conditions, humidity and quality of soil, comparability of data might thus be expected. Within the Chittagong region, there are 17 tea growing estates in Fatickchari, 3 in Rangunia and 1 in Banskhali. In this respect, Table 2 shows the measured radioactivity of ^226^Ra to be similar to the available literature data, while UTE values for ^232^Th and ^40^K show much lower values compared to the reported data in the literature. Moreover, radioactivity in the estate tea leaves from Rize in Turkey also show higher values than the present results from UTE. The activity concentration of the artificial ^137^Cs radionuclide for the UTE sample is shown to be below the detection limit (<0.4), while a substantial amount of ^137^Cs was reported in estate tea leaf samples from Turkey. Such a result indicates the contamination of sampling area via some known/unknown nuclear activities. There are no available studies on the radioactivity of marketed tea samples in Bangladesh, therefore studies on marketed tea leaf samples imported from abroad have been chosen for comparison. The average activity concentration of ^226^Ra and ^232^Th and ^40^K in tea leaf samples collected from the local market show greater values than the reported data from Turkey (except ^40^K) and Serbia. The fact that the concentrations of ^226^Ra, ^232^Th and ^40^K vary substantially across the various regions depends mainly on their concentrations in the bedrock from which the soil originates [65].

### 3.4. Committed Effective Dose, Threshold Consumption Rate and Carcinogenic Risk

**Figure 3:**
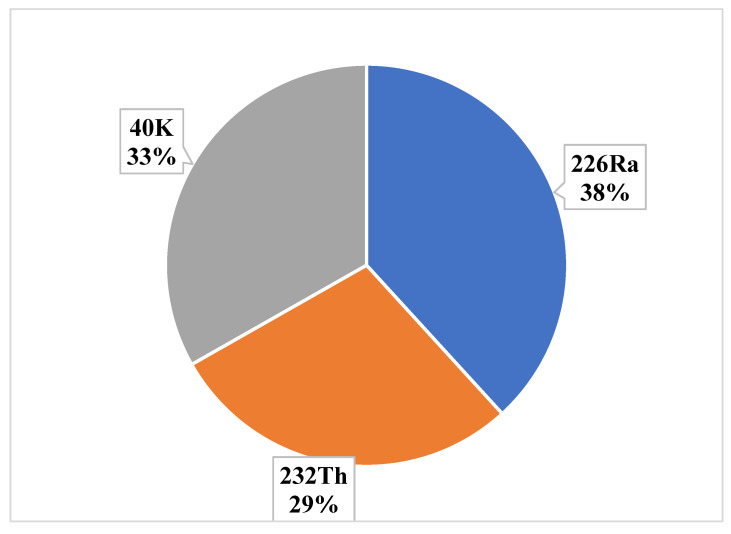
Dose contribution by individual radionuclides due to the consumption of tea leaves.

The calculated values of committed effective dose, threshold consumption rate and carcinogenic risk are shown in Table 3. The committed effective dose due to the intake of the studied radionuclides via tea consumption was found to be in the range of 4.7–5.6 µSv y^−1^ with a mean of 5.0 µSv y^−1^. This compares with an average worldwide ingestion dose of ^226^Ra and ^232^Th of 120 µSv y^−1^ and 170 µSv y^−1^ for ^40^K, making a total annual dose estimate of 290 µSv y^−1^ from the total diet [49]. The annual effective doses from the ingestion of tea leaves were found much lower than the limiting value recommended by UNSCEAR [49]. Note that the estimated 5.0 µSv y^−1^ is contributed to by only a single dietary element (here tea leaf), thus such a low value is not unexpected. However, the radiation risk via the cumulative consumption of tea leaf may not be totally negligible, because tea forms only a minor part of the total dietary habits [70,71]. Figure 3 shows the dose contribution due to the individual radionuclides. Among the studied radionuclides, ^226^Ra incurred the maximum dose (38%) followed by ^40^K (33%) and ^232^Th (29%). Exposure to radioactive materials, especially radium, over a prolonged time may result in an increased carcinogenic risk. In addition, higher doses of radium are found to have links with anemia, cataracts, reduction of bone growth, etc. [72].

The estimated mean threshold consumption rate for the studied tea leaf samples was found to be 88 kg/y (equivalent to 241g/d), an untenable value. This parameter indicates that the consumption of tea below the estimated value poses only a negligible radiological health hazard, while a greater rate than the calculated ones indicates enhanced radiological health risk.

Accordingly, the mean cumulative carcinogenic risk via tea leaf consumption (for a period of 50 years) was estimated at 1.3 × 10^−5^, significantly lower than the ICRP given cancer risk of 2.5 × 10^−3^, based on an annual effective dose limit of 1 mSv for the general population [52].

## 4. Conclusions

Activity concentrations of ^226^Ra, ^232^Th, ^40^K and ^137^Cs in soil and tea leaf samples collected from a large tea estate in the Chittagong region of Bangladesh were measured by HPGe γ-ray spectrometry. In addition, the most popular tea brands available in the local market were also measured to observe the effect of production processes. The transfer factor of radionuclides from soil to tea leaves was found to be less than 1, indicating the corresponding uptake by the tea plant to be insignificant. The estimated committed effective dose and the carcinogenic risk all show values far below the limiting ranges as suggested by various international bodies. Thus, the consumption of tea (at 4 g/day/person or two cups/day/person) produced from the Udalia Tea Estate provides an insignificant radiation risk to the health of the local populace. Considering a number of facts such as the non-availability of the literature data on TFs of the UTE tea leaves, the recommended limiting values for total dietary habits and that for the tea arising from this single entity, and that the radiation risk follows the linear no threshold model, the measured data can act as reference values for any future experimental or modelling studies for the protection of human health. 

## Figures and Tables

**Table 1:** Concentrations of ^226^Ra, ^232^Th, ^40^K and ^137^Cs in the analyzed soil and tea leaf samples (both fresh and marketed tea leaves) and calculated transfer factors from soil-to-tea leaf (fresh tea leaves from UTE (Udalia Tea Estate)).

Sampling Location	Sample Type	Activity Concentrations (Bq kg^−1^) Together with Uncertainty				Transfer Factor			
		^226^Ra	^232^Th	^40^K	^137^Cs	^226^Ra	^232^Th	^40^K	^137^Cs
Location-1	Garden Tea	5.7 ± 0.6	4.4 ± 0.5	190 ± 31	<0.4	0.13 ± 0.08	0.09 ± 0.05	0.69 ± 0.39	-
	Soil	45 ± 3	51 ± 1	275 ± 80	8.5 ± 1				
Location-2	Garden Tea	3.6 ± 0.7	3.2 ± 0.4	258 ± 29	<0.4	0.11 ± 0.08	0.05 ± 0.04	0.41 ± 0.13	-
	Soil	34 ± 9	63 ± 5	635 ± 35	7 ± 1				
Location-3	Garden Tea	5.7 ± 1.0	2.4 ± 0.5	175 ± 32	<0.4	0.15 ± 0.06	0.05 ± 0.01	0.55 ± 0.16	-
	Soil	37 ± 7	50 ± 13	391 ± 73	9 ± 1				
Location-4	Garden Tea	3.6 ± 0.6	5.8 ± 0.9	136 ± 22	<0.4	0.10 ± 0.03	0.09 ± 0.02	0.36 ± 0.13	-
	Soil	36 ± 7	65 ± 21	373 ± 73	4 ± 1				
Location-5	Garden Tea	4.1 ± 0.8	5.8 ± 1.1	132 ± 25	<0.4	0.10 ± 0.03	0.10 ± 0.04	0.54 ± 0.12	-
	Soil	42 ± 12	50 ± 19	245 ± 30	3 ± 1				
Mean						0.12 ± 0.08	0.08 ± 0.05	0.46 ± 0.35	-

**Table 2:** Average activity concentrations of ^226^Ra, ^232^Th, ^40^K and ^137^Cs in tea leaf samples from various countries compared with that from present work.

Sample Type	Countries	Activity Concentrations (Bq.kg^−1^) Together with Uncertainties				References
		^226^Ra	^232^Th	^40^K	^137^Cs	
Fresh tea leaf	UTE, Chittagong, Bangladesh	4.53 ± 0.62	4.31 ± 0.58	178 ± 28	<0.4	Present study
	Chittagong district, Bangladesh	5.34	10.07	429.91	Not measured	[66]
	Ramgarh, Bangladesh	3.20 ± 2.18	4.65 ± 1.76	625 ± 62.37	Not measured	[66]
	Kodala, Bangladesh	3.56 ± 0.69	27.22 ± 3.65	1243 ± 83.91	Not measured	[66]
	Chandpur Belgaon, Bangladesh	5.67 ± 2.16	12.41 ± 2.82	380 ± 62.06	Not measured	[66]
	Rize, Turkey	36.3 ± 6.1	23.1 ± 4.8	688.4 ± 18.3	20.9 ± 3.8	[67]
Market tea leaf	Mostafa-1	3.8 ± 0.4	6.0 ± 0.7	321 ± 35	<0.4	Present study
	Ceylon	4.3 ± 0.3	4.3 ± 0.5	244 ± 31	<0.4	
	Ispahani	4.0 ± 0.1	3.9 ± 0.5	159 ± 32	<0.4	
	Taza	5.4 ± 0.6	2.8 ± 0.6	141 ± 29	<0.4	
	Mostafa	4.3 ± 0.3	5.5 ± 0.8	183 ± 25	<0.4	
	Turkey-2 (Market tea)	0.9	2.7	501	-	[68]
	Serbia-1 (Market tea)	0.6−8.2	1.7−15.1	126−1243.7	-	[69]

**Table 3:** Calculated hazard parameters due to the consumption of studied tea leaves collected from the UTE, Chittagong, Bangladesh.

Sample	Annual Effective Dose (uSv/y)				Threshold Consumption Rate, kg/y	Lifetime Carcinogenic Risk
	^226^Ra	^232^Th	^40^K	Total		
TL-1	2.4	1.5	1.8	5.6	77	1.40 × 10^−5^
TL-2	1.5	1.1	2.4	5.0	88	1.24 × 10^−5^
TL-3	2.4	0.8	1.6	4.8	90	1.20 × 10^−5^
TL-4	1.5	1.9	1.3	4.7	93	1.17 × 10^−5^
TL-5	1.7	1.9	1.2	4.9	89	1.22 × 10^−5^
Mean	1.9	1.4	1.7	5.0	88	1.25 × 10^−5^

## Data Availability

All data are available in the manuscript.
